# Electronic Cigarette Usage and Psychological Distress: Insights from University Students Amidst the Armed Conflict in Southern Thailand

**DOI:** 10.3390/healthcare14101263

**Published:** 2026-05-07

**Authors:** Tharntip Sangsuwan, Chonnakarn Jatchavala, Bhawarun Akkaraprasit, Bunyisa Thaoun, Pariyawit Suwancharoen, Piyaphat Udompongpaiboon, Natnicha Ponintawong, Nontapat Lertnukkhid, Passaporn Chothikasatien, Irfan Saleh

**Affiliations:** 1 Department of Family and Preventive Medicine, Faculty of Medicine, Prince of Songkla University, Songkhla 90110, Thailand; be_med29@hotmail.com; 2 Department of Psychiatry, Faculty of Medicine, Prince of Songkla University, Songkhla 90110, Thailand; 3 Faculty of Medicine, Prince of Songkla University, Songkhla 90110, Thailand; bhawarun6run@gmail.com (B.A.); bunyisathaoun@gmail.com (B.T.); pond.sma@outlook.com (P.S.); piyaphat543@gmail.com (P.U.); ntp.natnicha@gmail.com (N.P.); nonlertnukkhid@gmail.com (N.L.); mim.passaporn@gmail.com (P.C.); ismiirfan@gmail.com (I.S.)

**Keywords:** attitudes, armed conflict, e-cigarette, mental health, student

## Abstract

**Background/Objectives**: Electronic cigarettes (e-cigarettes) are an important public health concern, particularly amongst young individuals. This study aimed to assess the attitudes and factors, including mental distress, that influence e-cigarette use amongst university students in Southern Thailand, including in armed conflict areas. **Methods**: A cross-sectional survey was conducted at the Prince of Songkla University across two campuses: Hat yai and Pattani. Data on demographics, attitudes towards e-cigarettes, usage patterns, and mental health (using the 21-item Depression, Anxiety, and Stress Scale) were collected and analysed using R software. **Results**: Amongst 901 participants (236 men and 665 women), the prevalence of e-cigarette use was 6.9%. Men (OR = 2.65; 95% CI = [1.28, 5.46]), tobacco users (OR = 97.56; 95% CI = [26.77, 355.66]), and those living alone (OR = 13.48; 95% CI = [1.21, 150.47]) were more likely to engage in e-cigarette use. Additionally, Islamic students reported lower usage rates than their Buddhist counterparts (OR = 0.32; 95% CI = [0.11, 0.9]). **Conclusions**: E-cigarette users exhibited higher scores on the depression, anxiety, and stress subscales than non-users. Smokers perceived e-cigarettes as a means of reducing tobacco consumption, alleviating stress, and enhancing their image. Moreover, non-smokers believed that e-cigarettes should be ‘illegal’ in Thailand.

## 1. Introduction

Electronic cigarettes (e-cigarettes or vapes) are designed to deliver nicotine in a vapour form [1]. They are predominantly marketed as smoking cessation aids and are potentially less harmful alternatives to traditional cigarettes [2]. E-cigarettes comprise a power source, a cartridge containing an atomiser and liquid solution, and a mouthpiece. The liquid, known as e-liquid or e-juice, is typically composed of glycerol, propylene glycol, water, flavours, and varying levels of nicotine [3], and e-cigarettes aim to mimic the smoking experience without using tobacco [4]. Notably, the World Health Organization has expressed concerns about the health risks associated with e-cigarettes, advocating for stricter regulations and warnings to prevent their use among non-smokers, youthful populations, and vulnerable groups [5,6]. Although few studies have examined the long-term health effects of e-cigarettes, evidence suggests that e-cigarettes can cause acute endothelial dysfunction, oxidative stress, and DNA damage [7]. Short-term risks include burns, injuries from device malfunctions, e-liquid toxicity, lung injuries associated with using e-cigarettes or vaping products, and pneumomediastinum [8]. Increasing evidence of these health risks has led to restrictions on e-cigarette sales in several U.S. states and in more than 98 countries [9].

Despite widespread awareness of the harms, e-cigarette use amongst children and young adults surged after the Food and Drug Administration approved a ‘heat-not-burn’ device in 2018 [10]. Even dental students across 11 countries displayed positive beliefs towards e-cigarettes, which affected their professional practices [11]. In Thailand, a study found that 28.7% of vocational students used e-cigarettes, of which 7.4% used them exclusively, and 21.3% used both e-cigarettes and traditional cigarettes [12].

The appealing design and vibrant packaging of e-cigarettes significantly influence young consumers’ purchasing decisions. Additionally, the various available flavours, such as fruity and candy, shape adolescents’ perceptions, leading them to underestimate the associated risks [13,14]. Young adults who use e-cigarettes often have a limited understanding of their potential dangers and a low perception of addiction. They consider e-cigarettes to be modern and more socially acceptable than traditional cigarettes [14,15]. Consequently, e-cigarette use amongst adolescents and young adults is rising globally, with market growth projected to reach 3.4% annually after 2023 [1].

In addition to attractive designs, stress plays a significant role in e-cigarette use amongst young people. A notable percentage of dental students reported using e-cigarettes to relieve stress and enhance pleasure [11]. Moreover, some young people who experience high levels of depression and anxiety may misinterpret their anxiety as stress, and some have reported using e-cigarettes and other substances as a means of self-medicating [13].

However, no study on e-cigarettes use amongst young people studying or living in the region of Southern Thailand’s insurgency has been conducted. The provinces affected by this rebellion include Pattani, Narathiwat, Yala, and limited areas of Songkhla. These regions are still regarded as volatile, and the Prince of Songkhla University is located in the centre (Pattani) of this region and in adjacent areas (Hat yai). A previous survey examining post-traumatic stress disorder (PTSD) symptoms amongst students at the Pattani and Hat yai campuses revealed a prevalence rate of 24.1%; furthermore, between 34.9% and 35.3% of participants reported persistent feelings of insecurity due to regional armed conflict prior to the COVID-19 pandemic [16]. The clinical presentation of PTSD is complex, often manifesting as intrusive thoughts and hyper-arousal, which may be misidentified as anxiety. Additionally, PTSD-related cognitive alterations and negative affect are frequently perceived as symptoms of stress or depression [16]. Consequently, interventions addressing substance use, including e-cigarette use, must account for the psychological distress of users and the potential for ‘self-medicating’ behaviours, particularly within these vulnerable populations [11,13].

This study aimed to explore the prevalence and factors related to e-cigarette use amongst university students. Specifically, it sought to examine levels of anxiety, depression, and stress amongst university students in the restive areas of Southern Thailand, their attitudes towards e-cigarettes, and the relationship between psychological distress (anxiety, depression, and stress) and e-cigarette usage. This study constitutes a component of a broader project investigation of e-cigarette use amongst university students in Southern Thailand, with a particular focus on those residing and studying within regions affected by armed conflict. The project aimed to develop tailored health promotion campaigns addressing the use of these novel tobacco products across university campuses located in the restive area of Southern Thailand.

## 2. Materials and Methods

### 2.1. Study Design and Participants

This observational cross-sectional study was conducted over 2 weeks, from March to April 2024. A Google Forms digital survey was distributed to undergraduate students enrolled at the Prince of Songkla University, at the Pattani campus, which is in a conflict-affected area of Southern Thailand, and the Hat yai campus, which is the university’s largest campus, situated in Songkhla, wherein many districts are also in conflict-affected areas. This study was approved by the ethics committee of the Faculty of Medicine at Prince of Songkla University (REC 67-094-91).

During the 2024 academic year, 19,173 undergraduate students attended the Hat Yai campus, and 7471 attended the Pattani campus. The minimum target sample size, determined using the R programme’s estimation of finite population proportion, was 670 students from both campuses. This estimation was based on a 95% confidence interval (CI) with a 5% margin of error, and an additional 10% was added to account for potential errors [11,12]. The inclusion criteria required participants to be students aged >18 years from the Hat yai and Pattani campuses who were proficient in the Thai language. Students who were experiencing severe mental or physical illnesses during the survey period were excluded. The severity of both types of illness was self-reported and identified as ‘ongoing treatment’, leading to exclusion from the participant group.

Convenience sampling was employed to select participants by selecting lecture classrooms from the following departments of three main schools: health-related sciences, non-health-related sciences, and social sciences. The lectures were officially scheduled for presentation during the survey period at both campuses.

### 2.2. Data Collection and Study Variables

The questionnaire comprised four sections: demographic information; smoking habits; attitudes towards e-cigarettes; and the 21-item Depression, Anxiety, and Stress Scale (DASS-21 [15]).

First, data were gathered on the participants’ sociodemographic characteristics, including age, sex, marital status, religion, academic year, faculty, place of residence, monthly income, perceptions of family relationships, and physical or mental health issues. Additionally, the history of drug use and smoking habits amongst close contacts was explored [17].

Second, the four-item smoking habits section assessed the respondents’ use of e-cigarettes and conventional cigarettes over the past 12 months, according to the DSM-5 criteria for substance-related disorders and based on prior studies in Thailand [11]

Third, the 10-item section examined participants’ perspectives on e-cigarettes. The Item Objective Congruence Index (IOC) was calculated and re-adjusted until the IOC value > 0.5. The first five questions assessed positive attitudes, whereas the last five questions assessed negative attitudes. Responses to questions about positive and negative attitudes towards e-cigarettes were indicated using a 5-point Likert scale: 1 (agree least strongly), 2 (agree less strongly), 3 (moderately agree), 4 (agree more strongly), and 5 (agree most strongly).

Fourth, the Thai version of the DASS-21 was employed, which included three subscales (depression, anxiety, and stress), each containing seven items. A 4-point Likert scale was used to measure severity levels: 0 (not applicable at all), 1 (somewhat applicable), 2 (considerably applicable), and 3 (very applicable). The DASS-21 cutoff points for the severity levels are presented in Table 1.

The first three sections of the study measurements involved creating a unified questionnaire in Thai. The content validity of the questionnaire was evaluated by three scholars before the questionnaire was finalised. The IOC index was determined for each question, and each question was adjusted until the item objective congruence value exceeded 0.5.

### 2.3. Data Analysis

Data were compiled using Microsoft Excel. All data were processed and analysed using R (version 4.1.3). Normality was assessed using the Shapiro–Wilk Test. Descriptive statistics are reported as means, frequencies, and percentages. The chi-square test and Fisher’s exact test were used to assess the significance of comparisons between the two groups. Spearman correlations and logistic regression were used for association analysis.

## 3. Results

### 3.1. Demographic Information and E-Cigarette Use Amongst University Students in Southern Thailand

Based on the inclusion criteria, 942 students were included, and 41 students were excluded (Table 2; 236 men, 665 women). Most participants identified as single (84.0%), the majority identified as Buddhist (50.8%), and a large majority reported being both physically and mentally healthy (90.5% and 97.9%, respectively) without any history of substance abuse (96.2%). Most participants were first-year students (60.4%), studied social sciences (49.5%), had GPAs > 3.0 (63.1%), and resided in a university dormitory (56.8%). Approximately 20.9% were from areas affected by the Southern Thailand insurgency, whereas most participants were from other regions of Southern Thailand (73.0%). Their average income ranged between 5000 and 10,000 baht (94.0%), and they generally perceived their family relationships as positive (90.4%). However, 53.4% reported being surrounded by smokers (both tobacco and e-cigarette users), predominantly family members (29.2%), despite reporting no history of tobacco use (96.6%).

The results in Table 2 reveal a statistically significant variation in the prevalence of e-cigarette use amongst students based on several criteria: sex, marital status, religion, faculty affiliation, domicile, place of residence, income, GPA, tobacco use, close contact with smokers, and other substance use.

### 3.2. Factors Related to E-Cigarette Use Among University Students in the Restive Areas of Southern Thailand

Table 3 presents the factors influencing the utilisation of e-cigarettes. The likelihood of e-cigarette usage was 2.65 times greater amongst men (95% CI = [1.28, 5.46]) compared to women. Additionally, students who used tobacco had a considerably higher probability of using e-cigarettes (97.56 times higher) than those who did not (95% CI = [26.77, 355.66]). Furthermore, the likelihood of a student using e-cigarettes was significantly greater for those residing in apartments and living alone (by a factor of 2.44, 95% CI = [1.11, 5.35], and 13.48, 95% CI = [1.21, 150.47], respectively). Students with complicated marital statuses or ‘situationships’ were 2.01 times more likely to use e-cigarettes than those who were single (95% CI = [0.62, 6.47]). Additionally, students with a GPA below 2.00 exhibited 3.56 times higher rates of e-cigarette use than those with a GPA above 3.00 (95% CI = [0.54, 23.46]).

Notably, a significantly lower likelihood of e-cigarette usage was noted amongst participants who identified as Islamic than those who identified as Buddhist (OR = 0.32; 95% CI = [0.11, 0.9]).

### 3.3. Attitudes and Perceptions Towards E-Cigarette Use Amongst University Students in the Restive Areas of Southern Thailand

Table 4 presents the relationship between e-cigarette usage and perceptions amongst these students by comparing non-users with users. Users generally agreed that e-cigarettes helped reduce smoking, were less harmful, and effectively reduced stress. Conversely, users frequently or strongly agreed with these positive statements (*p* < 0.001 for Items 1–5).

Both non-users and users largely or strongly agreed that e-cigarettes were addictive, should remain illegal in Thailand, and were harmful to individuals’ physical and mental health and to children and pregnant women. However, compared with users, non-users tended to agree significantly more that e-cigarettes should remain illegal in Thailand (*p* < 0.001). These results suggest that positive attitudes and perceptions towards e-cigarettes may be related to participant usage (Table 4).

### 3.4. Relationship Between E-Cigarette Usage and Students’ Depression, Anxiety, and Stress Levels (DASS-21)

Table 5 presents the relationship between the DASS-21 score and e-cigarette usage. Individuals in the e-cigarette user group exhibited notably elevated scores across all three subscales (depression, anxiety, and stress) compared with non-users (*p*-values < 0.001, 0.0006, and 0.0010, respectively).

**Figure 1:**
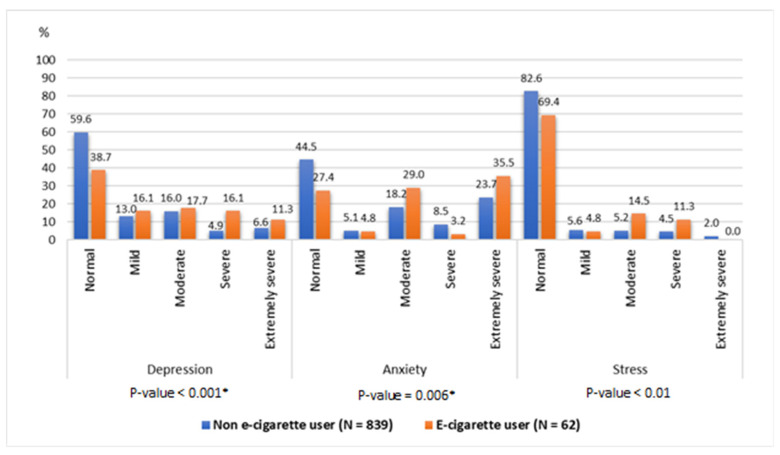
Association between e-cigarette usage and students’ depression, anxiety, and stress (DASS-21) by severity. * *p*-value ≤ 0.05 by Fisher’s exact test.

Based on the DASS-21 scoring guidelines, Figure 1 presents students’ frequency of experiencing depression, anxiety, and stress at different severity levels, classified as normal, mild, moderate, severe, and extremely severe. This study found the prevalence of depression, anxiety, and stress to be 41.8%, 32.6%, and 18.3%, respectively. The results suggested that e-cigarette use was significantly associated with stress (*p* = 0.010), anxiety (*p* = 0.006), and depression (*p* < 0.001).

## 4. Discussion

This cross-sectional study found a low occurrence of e-cigarette usage amongst university students in the armed-conflict areas of Southern Thailand. The prevalence of e-cigarette usage across both campuses was 6.9%, while conventional cigarette use was recorded at 3.3%. When compared to previous research conducted among Thai students prior to the COVID-19 pandemic [18], which aligned with a study conducted amongst high school students in Indonesia [17], these findings indicate a rising trend in e-cigarette consumption alongside a concurrent decline in traditional tobacco use.

Moreover, our findings reveal a strong association between several risk factors and e-cigarette use in the armed-conflict areas of Southern Thailand. These risk factors include the male sex, a history of tobacco use, and place of residence. This finding is consistent with that of a previous report from China [19]. One explanation for this may be that compared with female adolescents, male adolescents perceive e-cigarettes as less detrimental than traditional cigarettes [20]. Additionally, this study revealed that conventional tobacco users were significantly more likely to use e-cigarettes than non-tobacco users [20], which aligns with the findings of the seven articles mentioned in a previous systematic review conducted in Southeast Asia [21]. Furthermore, students residing in apartments and those living alone were shown to be more likely to use e-cigarettes than those residing in dormitories. This finding differs from previous studies. For example, a study conducted in the United States indicated that college students residing in university dormitories may be more influenced by their peers who use e-cigarettes [22].

Compared with Buddhist students in lower Southern Thailand, Muslim students exhibited a notably lower likelihood of engaging in e-cigarette use. Although no Islamic perspective was examined amongst these individuals, this phenomenon appears to align with previous studies conducted in Malaysia, which revealed that most smokers were aware of the ‘Fatwa’ prohibiting smoking in Islam [23].

Regarding the participants’ attitudes towards e-cigarettes, e-cigarettes were perceived as more accessible than traditional cigarettes. This aligns with findings from a previous study conducted in 2023 that revealed that a significant portion of Thai participants commonly displayed negative attitudes towards e-cigarettes and indicated that e-cigarette users tended to have more misunderstandings than non-users [24]. To curb the proliferation of new e-cigarette consumers, undergraduate students should be encouraged to cultivate a precise understanding of e-cigarette usage, focusing on health risks and the insufficiency of evidence supporting e-cigarettes as a means of tobacco cessation [25].

Our findings also reveal a significant correlation between the DASS-21 scores and e-cigarette use. Notably, e-cigarette users obtained higher scores on all three subscales (depression, anxiety, and stress) than non-users. This may indicate that struggling with mental health issues is significantly associated with e-cigarette use, and it may be linked to some less visible psychological disorders such as PTSD [16,26]. This finding is consistent with a nationwide study that observed a bidirectional association between e-cigarette use and mental illness amongst young adults [26]. According to neurobiological studies, individuals with depression may resort to smoking as a form of ‘self-medication’ to enhance the immediate transport of nicotine to brain cells and, thereby, alleviate symptoms associated with depression. However, prolonged nicotine use can impair long-term monoamine function, potentially worsening or prolonging depression and leading to addiction [25]. In addition to psychological distress, nicotine consumption is also associated with significant physiological consequences from DNA damage and oxidative stress; policymakers, educators, and users should be aware of both its physical and psychological effects, as smoking e-cigarettes is an ‘unhealthy habit’ [3,7,8].

The alleviation of stress is a common reason given for using e-cigarettes. A multivariate logistic regression analysis in a previous study indicated that the relationship between stress and e-cigarette use was significant; some research even suggests that the belief that e-cigarettes reduce stress may be a myth [27]. However, the findings of the previous study prioritised stress reduction and promoted healthy coping strategies to prevent the initiation of vaping as a common ‘stress-reliever’ in Thai young adults [26,27].

Although several studies found no direct connection between anxiety and e-cigarette use, the results of this study indicate that anxiety is related to the perceived advantages of e-cigarettes. Given these mixed results, unlike stress and depression, the relationship between anxiety and vaping might not be straightforward. Consequently, more studies are required to explore the relationship between anxiety and e-cigarette use, and psychological interventions should be appropriately tailored to students managing psychological distress and vaping. Furthermore, this study calls for additional training for both higher education and medical personnel to improve their capacity to provide the necessary counselling to smokers at universities [28].

This study had several limitations. First, some affiliated faculty members had smaller sample sizes than others, which may have led to results that do not fully reflect the overall population. Because mental and physical illness were self-reported and could be misinterpreted, their exclusion may have influenced the results, confounding the target population of healthy individuals; furthermore, in addition to the participants’ peer pressure and lifestyle (living in an armed conflict situations), attitude towards other addictive substances and coping strategies towards the ongoing violent situations could affect e-cigarette use [29]. Furthermore, the stigma or reluctance associated with openly discussing e-cigarette use owing to social and cultural influences should be acknowledged, particularly because this activity is illegal in Thailand. Asking about recent usage (i.e., use in the past 30 days) may be more strongly reported by participants.

## 5. Conclusions

The prevalence of e-cigarette use amongst university students in the restive areas of Southern Thailand in this study was 6.9%. A significant association was observed between e-cigarette use and mental distress, particularly depression, anxiety, and stress. Participants who had utilised e-cigarettes within the previous 12 months were significantly more likely to dispute the assertion that these products pose risks to physical and mental health compared to conventional cigarettes. Furthermore, these individuals expressed the belief that e-cigarettes facilitate smoking cessation and offer a more favourable social image than does traditional tobacco. Conversely, non-users, who comprised the vast majority of the study population, maintained the conviction that e-cigarettes should remain prohibited under Thai law. Therefore, when educational administrators and policymakers who are concerned about e-cigarette use amongst students create smoking cessation campaigns, such campaigns should specifically target students in consideration of their mental health statuses and attitudes towards e-cigarettes.

## Figures and Tables

**Table 1:** The cutoff scores for the 21-item Depression, Anxiety, and Stress Scale (DASS-21) by severity [15].

	Depression	Anxiety	Stress
Normal	0–9	0–7	0–14
Mild	10	8	15
Moderate	14	10	19
Severe	21	15	26
Extremely severe	28	20	34

**Table 2:** University students’ demographic information related to e-cigarette usage (n = 901).

Variable	Total	Non-E-Cigarette User	E-Cigarette User	*p*-Value
	n = 901 (100%)	n = 839 (93.1%)	n = 62 (6.9%)	
Sex				<0.001 ^a^*
Male	236 (26.2)	199 (23.7)	37 (59.7)	
Female	665 (73.8)	640 (76.3)	25 (40.3)	
Marital status				<0.001 ^a^*
Single	757 (84.0)	714 (85.1)	43 (69.4)	
Married	0 (0)	0 (0.0)	0 (0.0)	
In relationship	104 (11.5)	93 (11.1)	11 (17.7)	
Situationship	40 (4.5)	32 (3.8)	8 (12.9)	
Religion				<0.001 ^b^*
Buddhism	458 (50.8)	410 (48.9)	48 (77.4)	
Islam	435 (48.3)	423 (50.4)	12 (19.4)	
Other	8 (0.9)	6 (0.7)	2 (3.2)	
School of study				0.030 ^a^*
Health-related sciences	195 (21.6)	184 (21.9)	11 (17.7)	
Non-health-related sciences	260 (28.9)	233 (27.8)	27 (43.5)	
Social sciences	446 (49.5)	422 (50.3)	24 (38.7)	
Domicile				0.048 ^a^*
Other Southern regions	658 (73.0)	608 (72.5)	50 (80.6)	
Armed-conflict areas	189 (21.0)	183 (21.8)	6 (9.7)	
Other regions of Thailand	54 (6.0)	48 (5.7)	6 (9.7)	
Monthly income (baht)				<0.001 ^b^*
<5000	487 (54.1)	470 (56.0)	17 (27.4)	
5000–10,000	361 (40.1)	327 (39.0)	34 (54.8)	
10,001–50,000	50 (5.5)	39 (4.6)	11 (17.7)	
>50,000	3 (0.3)	3 (0.4)	0 (0.0)	
Perceived family relationship				0.124
Good	815 (90.5)	763 (90.9)	52 (83.9)	
Bad	23 (2.5)	21 (2.5)	2 (3.2)	
Neutral	63 (7.0)	55 (6.6)	8 (12.9)	
Grade point average				0.005 ^a^*
<2.0	209 (23.2)	187 (22.3)	22 (35.5)	
2.0–2.5	99 (11.0)	90 (10.7)	9 (14.5)	
2.5–3.0	24 (2.7)	20 (2.4)	4 (6.5)	
>3.0	569 (63.1)	542 (64.6)	27 (43.5)	
History of physical disorders				0.405
Yes	85 (9.4)	81 (9.7)	4 (6.5)	
No	816 (90.6)	758 (90.3)	58 (93.5)	
History of psychiatric disorders				1.000
Yes	882 (97.9)	821 (97.9)	61 (98.4)	
No	19 (2.1)	18 (2.1)	1 (1.6)	
Tobacco use				<0.001 ^a^*
No	871 (96.7)	835 (99.5)	36 (58.1)	
Yes	30 (3.3)	4 (0.5)	26 (41.9)	
Being around smokers				<0.001 ^a^*
No	420 (46.6)	407 (48.5)	13 (21.0)	
Yes	481 (53.4)	432 (51.5)	49 (79.0)	
Surrounded by people who smoke (n = 481)				<0.001 ^b^*
Couple	24 (26.7)	20 (4.6)	4 (8.2)	
Family member(s)	266 (29.2)	258 (59.3)	8 (16.3)	
Friend(s)	157 (17.4)	120 (27.6)	37 (75.5)	
Senior(s) or junior(s)	37 (40.6)	37 (8.5)	0 (0.0)	
History of substance use				0.005 ^b^*
No	876 (97.2)	820 (97.7)	56 (90.3)	
Yes	25 (2.8)	19 (2.3)	6 (9.7)	
Place of residence				<0.001 ^b^*
Friend(s) or couple(s)	23 (2.5)	21 (2.5)	2 (3.2)	
Dormitory	517 (57.4)	498 (59.4)	19 (30.6)	
Apartment	228 (25.3)	195 (23.2)	33 (53.2)	
Living alone at home	5 (0.6)	2 (0.2)	3 (4.8)	
Home with family	128 (14.2)	123 (14.7)	5 (8.1)	

* *p*-value ≤ 0.05, a = chi-square test, and b = Fisher’s exact test.

**Table 3:** Associated factors of e-cigarette use amongst university students in the armed-conflict areas of Southern Thailand.

Variable	Crude Odds Ratio (95% CI)	Adjusted Odds Ratio (95% CI)
Sex		
Male	4.76 (2.8, 8.1)	2.65 (1.28, 5.46)
Female	1.00 (reference)	1.00 (reference)
Marital Status		
Single	1.00 (reference)	1.00 (reference)
In relationship	1.96 (0.98, 3.94)	1.1 (0.43, 2.79)
Situationship	4.15 (1.8, 9.55)	2.01 (0.62, 6.47)
Religion		
Buddhism	1.00 (reference)	1.00 (reference)
Islam	0.24 (0.13, 0.46)	0.32 (0.11, 0.9)
Other	2.85 (0.56, 14.5)	1.03 (0.08, 14.08)
School of Study		
Health-related science	1.05 (0.5, 2.19)	1.18 (0.44, 3.19)
Non-health-related science	2.04 (1.15, 3.61)	1.05 (0.46, 2.41)
Social science	1.00 (reference)	1.00 (reference)
Domicile		
Southern Thailand	2.51 (1.06, 5.94)	1.07 (0.32, 3.57)
The restive areas of Southern Thailand	1.00 (reference)	1.00 (reference)
Other regions of Thailand	3.81 (1.18, 12.35)	0.6 (0.1, 3.6)
Monthly income (baht)		
<5000	1.00 (reference)	1.00 (reference)
5000–10,000	2.87 (1.58, 5.23)	1.56 (0.68, 3.56)
10,001–50,000	7.8 (3.41, 17.81)	1.99 (0.53, 7.47)
>50,000	0 (0, inf)	0 (0, inf)
Grade point average		
<2.0	4.01 (1.28, 12.57)	3.56 (0.54, 23.46)
2.0–2.5	2.01 (0.91, 4.41)	1.55 (0.48, 5.08)
2.5–3.0	2.36 (1.31, 4.25)	2.14 (0.97, 4.69)
>3.0	1.00 (reference)	1.00 (reference)
Tobacco use		
No	1.00 (reference)	1.00 (reference)
Yes	150.76 (49.97, 454.86)	97.56 (26.77, 355.66)
Being around smokers		
No	1.00 (reference)	1.00 (reference)
Yes	3.55 (1.9, 6.64)	1.73 (0.81, 3.69)
History of substance use		
No	1.00 (reference)	1.00 (reference)
Yes	4.62 (1.78, 12.04)	2.07 (0.37, 11.65)
Place of residence		
Lived with friend(s) or couple(s)	2.5 (0.55, 11.43)	2.92 (0.42, 20.35)
Living alone	39.32 (6.2, 249.27)	13.48 (1.21, 150.47)
University dormitory	1.00 (reference)	1.00 (reference)
Apartment	4.44 (2.46, 7.99)	2.44 (1.11, 5.35)
Home	1.07 (0.39, 2.91)	1.31 (0.42, 4.09)

odds ratio = OR and confidence interval = CI by logistic regression.

**Table 4:** Attitudes and perceptions regarding e-cigarette use amongst the university students.

Question	Non-User (n = 839)	User (n = 62)	*p*-Value
Do you think that e-cigarettes help reduce smoking?	2 [1,3]	3 [2,4]	<0.001 *
Do you think that e-cigarettes reduce the harm of cigarette smoke to people around you compared with cigarette smoke?	1 [1,2]	2 [1,3]	<0.001 *
Do you think that e-cigarettes are less harmful to smokers’ health than cigarettes?	1 [1,3]	2.5 [1,4]	<0.001 *
Do you think that smoking e-cigarettes gives a better public image than smoking cigarettes?	1 [1,2]	2 [1.25,3]	<0.001 *
Do you think that e-cigarettes can be used to reduce stress effectively?	1 [1,3]	3 [2.25,4]	<0.001 *
Do you understand that e-cigarettes are addictive?	4 [3,5]	4 [3,5]	0.897
Do you hold the opinion that e-cigarettes should remain illegal?	4 [2,5]	3 [2,3]	<0.001 *
Do you understand that e-cigarettes are harmful to both physical and mental health?	4 [3,5]	4 [3,5]	0.25
Do you understand that e-cigarettes are harmful to children and pregnant women?	5 [4,5]	5 [4,5]	0.976
Do you feel that e-cigarettes are more accessible and easier to buy than cigarettes?	3 [2,4]	3 [3,4]	0.904

* *p*-value ≤ 0.05 by Wilcoxon rank-sum test.

**Table 5:** E-cigarette use and its association with depression, anxiety, and stress amongst students (DASS-21).

Psychological Distress	Mean Score	Mean Score	*p*-Value
	Non-E-Cigarette User	E-Cigarette User	
Depression	3 [0,7]	6 [2.25,11]	<0.001 *
Anxiety	5 [0,9]	7 [3,12]	0.006 *
Stress	2 [0,6]	3.5 [1,10.5]	0.010 *

* *p*-value ≤ 0.05 by Wilcoxon rank-sum test.

## Data Availability

As the datasets in this study are highly sensitive, they are not publicly available. However, reasonable requests will be considered through contact with the first author or corresponding author.

## Abbreviations

The following abbreviations are used in this manuscript:

PTSD	Post-traumatic stress disorder
DSM-5	Diagnostic and Statistical Manual of Mental Disorders, Fifth Edition
GPA	Grade point average
OR	Odds ratio
CI	Confidence interval
DASS-21	21-item Depression, Anxiety, and Stress Scale
